# *TP53* Mutations in Mantle Cell Lymphoma: From Backup to Game Changer

**DOI:** 10.3390/jcm14238480

**Published:** 2025-11-29

**Authors:** Maria Elena Carazzolo, Alessia Moioli, Carlo Visco

**Affiliations:** 1 University Laboratory for Medical Research (LURM), Department of Medicine, University of Verona, 37134 Verona, Italy; mariaelena.carazzolo@univr.it; 2 Centro Ricerche Cliniche s.r.l, 37134 Verona, Italy; alessia.moioli@crc.vr.it; 3 Hematology and Bone Marrow Transplant Unit, Department of Engineering for Innovation Medicine, Section of Biomedicine, University of Verona, 37134 Verona, Italy

**Keywords:** mantle cell lymphoma, *TP53*, NGS, standardization, prognosis, precision medicine

## Abstract

Mantle cell lymphoma (MCL) is an aggressive subtype of non-Hodgkin lymphoma (NHL) whose clinical course is largely shaped by molecular and biological features. Among the most impactful prognostic markers, *TP53* mutations have emerged as critical determinants of treatment resistance since their first identification in MCL in 1996. Regardless of the detection method, *TP53* mutations have been consistently associated with primary refractoriness to chemoimmunotherapy and significantly reduced overall survival. In this perspective, we explored recent advances in applying integrated-omics approaches to assess *TP53* status. Despite its prognostic value, routine testing for *TP53* at diagnosis remains uncommon, hindered by the lack of standardized protocols and costs for Next-Generation Sequencing (NGS), and the suboptimal reliability of immunohistochemistry (IHC) as a surrogate. This gap between research evidence and clinical practice represents a critical barrier to risk-adapted therapy. The broad implementation of standardized and accessible genomic techniques is essential to identify patients who deserve a personalized therapeutic approach. Several clinical trials have recently explored alternative chemo-free or targeted regimens specifically tailored to *TP53*-mutated patients (i.e., NCT03824483, NCT03567876), with promising results. This risk-adapted approach reflects a paradigm shift in MCL management, emphasizing the need for early molecular risk assessment to guide treatment decisions. In this scenario, *TP53* mutations are no longer supporting actors, but a game-changer for the prognosis and treatment of patients with MCL.

## 1. Introduction

Mantle cell lymphoma (MCL) is a rare non-Hodgkin lymphoma (NHL) of mature B cells, accounting for 4–7%. It is commonly divided into two distinct groups: aggressive classic MCL (cMCL) and indolent leukemic non-nodal MCL (nnMCL) [1], which display a heterogeneous clinical course. The indices regularly adopted to discriminate the aggressiveness of MCL are: the Mantle Cell Lymphoma International Prognostic Index (MIPI) [2,3,4,5]; the morphologic features (classic vs. blastoid) [6,7,8,9]; and the evaluation of tumor kinetics (i.e., Ki67 expression).

A complex genomic landscape also characterizes MCL. The common genetic hallmark is the presence of t(11;14), which juxtaposes the cyclin D1 gene (*CCND1*) with the immunoglobulin heavy chain locus (*IGH*), leading to overexpression of cyclin D1 [3,4,10,11]. Moreover, MCL presents a high degree of genetic heterogeneity, which poses a significant challenge in identifying possible predictive and prognostic biomarkers [12,13]. A set of “driver” genes has been widely described, which are strongly involved in MCL pathogenesis, such as *ATM*, *TP53*, *NOTCH1/2*, *KMT2D*, *CCND1*, and *HNRNPH1* [1,11,12,14]. Secondary genomic events are also well-known, with losses at 17p, 13q33-q34, 1p22, 11q22-q23, 6q, 13q14 (*RB1*), 9p21 (*CDKN2A/B*), 9q22q31, and 10p15-p13, and gains at 3q25-q29, 18q21-q22 (*BCL2*), and 12q13 (*CDK4*) [15] being the most frequent.

*TP53*, a tumor-suppressor gene, is one of the major players in cancer development, holding a strong role in the pathogenesis and therapy resistance of several tumors, including MCL [2,11]. Indeed, *TP53*, encoded on the short arm of chromosome 17, is also known as the “guardian of the genome” due to its role in regulating multiple cellular responses to stress stimuli through various mechanisms, including cell-cycle arrest, apoptosis, and senescence [16]. The dysregulation of *TP53* leads to two main groups of mutations: loss-of-function (LOF) and gain-of-function (GOF) mutations. *TP53* mutations occur in 5–20% of MCL cases at disease presentation, and their identification is becoming more and more important in the diagnostic/prognostic work-up of MCL patients at the time of diagnosis [17,18]. For instance, the missense mutations, classified as GOF, account for around 30% of all the mutations [19], principally occurring in the so-called hotspots of p53, leading to the activation or inactivation of several genes involved in cell-cycle regulation and survival, such as *ATM*, *CDKN2A*, and *NOTCH1*. The co-occurrence of these alterations leads to an increase in genomic instability, a more aggressive and accelerated tumor progression related to the blastoid variant of MCL [20], and a tendency to treatment resistance [21]. Indeed, *TP53* mutations may increase the expression of MDR1 (multidrug resistance gene 1), thereby enhancing the possibility of displaying various chemo-resistant mechanisms [19]. Furthermore, the *TP53* perturbation could have an impact on the BCR/PI3K/AKT signaling. The B-cell receptor (BCR) engagement initiates the formation of a complex signalosome, which phosphorylates a series of protein tyrosine kinases (PTKs), thereby regulating the physiological fate of B-cells, including survival and proliferation [22,23]. The loss of *TP53* leads to a highly inflammatory and immunosuppressive tumoral microenvironment, activating the NF-κB pathway (directly linked to BCR signaling) [19,24], which allows for possible immune escape and increases the likelihood of treatment resistance, for example, to Bruton kinase inhibitors (BTKi) [25], targeted therapies against CD20 and *BCL2* [26,27], and possibly to CAR-T [17]. To overcome this issue, some trials have attempted different combinations of drugs (NCT06482684, NCT05495464, NCT05861050). However, it is desirable to increase the research in the identification of new potential druggable targets in MCL p53-mutated cells.

**Figure 1:**
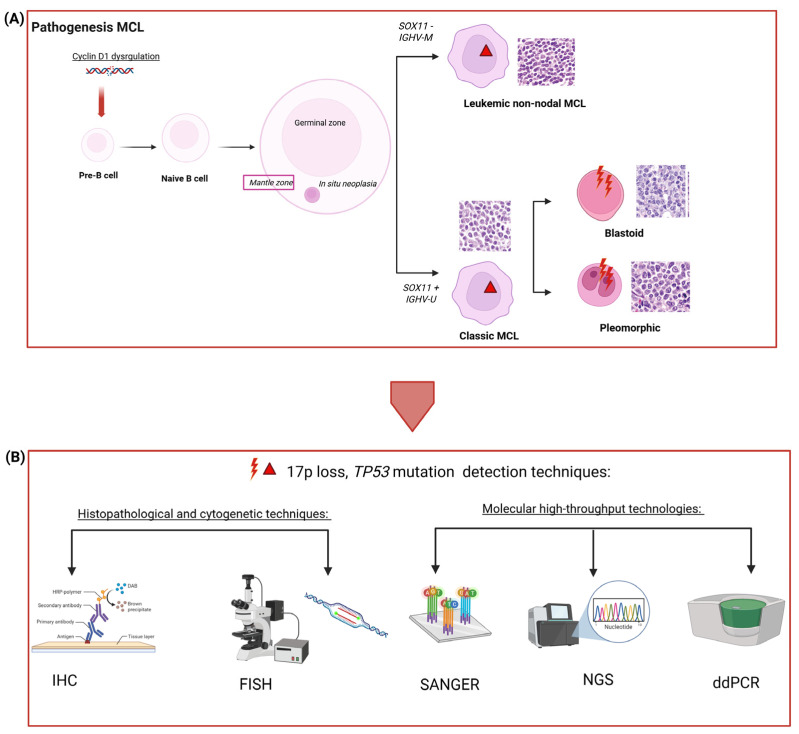
(**A**) shows the pathogenesis of MCL. A morphologic evaluation of the tumor-associated B-cells is commonly performed to classify MCL as indolent (leukemic-non-nodal morphology) or aggressive (classic morphology subdivided into two even more aggressive shapes: blastoid and pleomorphic). (**B**) represents the technologies (IHC, FISH, Sanger Sequencing, NGS, or ddPCR) available to determine the *TP53* status (mutation and/or aberration) for prognostication or risk prediction in patients.

The use of different techniques supported the detection of *TP53* mutations and aberrations with different accuracy and sensitivity, namely (I) immunohistochemistry (IHC), through p53 expression indirectly predicts *TP53* mutations; (II) Fluorescence In Situ Hybridization (FISH), a cytogenetic technique employed to detect *TP53* aberrations, and multiple molecular analysis of DNA conducted through (III) Sanger sequencing, (IV) Next-Generation Sequencing (NGS), and (V) droplet digital PCR (ddPCR) which are able to detect mutations, hotspots and copy number variations (CNVs), and deletions of *TP53* with different degrees of accuracy (Figure 1, Table 1). However, a comprehensive comparative analysis assessing which technique presents the highest sensitivity, reproducibility, and accuracy of *TP53* mutational status detection is still lacking [28].

In this prospective study, we review the clinical studies showing the prognostic relevance of *TP53* mutations in MCL, their impact on therapeutic decision-making [29], and the technologies used in most diagnostic and research laboratories.

## 2. Clinical Evidence of *TP53*’s Role in Resistance to Treatments and Poor Prognosis

*TP53* was identified as a strong and independent prognostic factor associated with inferior outcomes and treatment resistance in several prospective studies (see Table 2).

In younger, fit patients eligible for high-dose chemotherapy and autologous stem cell transplantation, *TP53* mutations confer a particularly dismal prognosis. In the pivotal study by Delfau-Larue et al., the addition of rituximab and high-dose cytarabine failed to overcome the adverse impact of *TP53* and *CDKN2A* deletions [30]. The Nordic MCL2 and MCL3 trials confirmed that patients harboring *TP53* mutations had markedly shorter progression-free survival (PFS) and overall survival (OS) compared to *TP53* wild-type (WT) cases (median OS 1.8 vs. >10 years; median PFS 0.9 vs. >10 years) [10].

In older patients treated with bendamustine and rituximab (BR), similar trends have been observed with *TP53*-mutated patients characterized by inferior survival, highlighting the persistence of the negative prognostic impact, even when following less intensive regimens [31]. Recent therapeutic strategies have focused on incorporating targeted agents earlier in the treatment algorithm. For instance, in the TRIANGLE trial, adding ibrutinib to intensive chemoimmunotherapy reduced the risk of failure in patients with high p53 expression by IHC. However, *TP53* mutational status was not reported [32]. Conversely, in the SHINE and ECHO trials, the addition of BTK inhibitors (ibrutinib or acalabrutinib) to BR did not significantly impact an outcome, and *TP53*-mutated patients continued to fare poorly [33,34].

Chemo-free combinations may represent a more promising approach in this setting. The combination of ibrutinib and rituximab in elderly patients with newly diagnosed MCL in the ENRICH trial yielded high overall response rates, yet those with *TP53* aberrations had inferior OS and a trend toward poorer outcomes [35]. More recent chemo-free triplet combinations have demonstrated greater efficacy. In the BoVen study, zanubrutinib, obinutuzumab, and venetoclax *TP53*-mutated patients achieved an overall response rate (ORR) of 86%, a complete response (CR) rate of 64%, and a 2-year PFS of 72% [36]. However, due to the small size of the *TP53*-mutated cohort and the relatively short follow-up, further studies with larger patient populations and longer follow-up are needed to confirm these findings. Similarly, the SYMPATICO trial combining ibrutinib and venetoclax reported an ORR of 84%, CR rate of 57%, median PFS of 20.9 months, and OS of 47.1 months in this population—a significant improvement compared to historical data [37]. The VR-BAC trial represented a prospective study stratifying treatment based on biological risk, including *TP53* mutations. The results suggest an enhanced activity of R-BAC plus venetoclax in high-risk subgroups, although outcomes remained poor in patients with concurrent blastoid morphology and *TP53* mutations [38].

As for cellular therapies, particularly CD19-directed CAR T-cells, have also shown promise. In the ZUMA-2 trial, brexucabtagene autoleucel demonstrated a 1-year PFS of 61% and a CR rate of 83%, with exploratory analyses suggesting benefits as well in TP53-mutated patients, although the numbers are small [39]. The TARMAC study, combining CAR-T and ibrutinib, achieved deep responses, including MRD negativity, regardless of *TP53* status [40]. More recently, a three-year follow-up of KTE-X19 by Wang et al. confirmed the durability of responses across most subgroups, including those with *TP53* mutations. Although the precise outcomes for *TP53*-mutated patients were not separately reported, the authors noted that this high-risk subgroup appeared to derive clinical benefit, with ongoing responses observed. Although clinical benefit was observed in this high-risk population, the number of *TP53*-mutated patients was limited, and the exploratory nature of the subgroup analysis warrants caution [41].

A recent multicenter analysis consistently reported *TP53* mutations in 51% of patients relapsing after CAR-T, reinforcing that *TP53* alterations contribute substantially to resistance and poor clinical outcomes [17]. Likewise, the bispecific antibodies, such as glofitamab, are also being evaluated. In the original phase I/II study on glofitamab in relapsed/refractory (R/R) MCL, five patients with *TP53* mutations were reported, of whom three (60%) achieved a complete response (CR) [32]. The updated data presented at ASH 2024 expanded this subgroup to nine patients with high-p53 expression (>50%), showing a CR rate of 67% compared to 76% in patients with a p53 expression below 50%, indicating a slightly lower response in the high-p53 subgroup. Survival analyses combining all high-risk features (including p53 expression > 50%, Ki-67 > 50%, and blastoid morphology) demonstrated that these patients had durable responses, with a median duration of CR of 21.5 months versus 19.2 months in patients without high-risk features [33]. Despite the encouraging activity of glofitamab in this difficult-to-treat subgroup [42,43], the small sample size and combined analysis of multiple high-risk markers warrant cautious interpretation, and further studies with larger, genetically defined cohorts are needed to confirm these findings. Building on the promising results from chemo-free combinations, as well as CAR-T and bispecific antibody studies in *TP53*-mutated MCL, several ongoing trials are now exploring risk-adapted strategies, specifically targeting high-risk patients. The CARMAN phase II trial is investigating early intensification with brexucabtagene autoleucel after abbreviated induction in patients with *TP53* mutation or p53 overexpression (NCT06482684). Another pilot study, WINDOW-3, is evaluating the combination therapy with acalabrutinib and rituximab as a “window” followed by CAR-T in newly diagnosed high-risk MCL (NCT05495464). Moreover, another phase I/II trial is testing the combination of glofitamab, venetoclax, and lenalidomide (NCT05861050) in front-line high-risk MCL (including *TP53*-mutated).

Emerging evidence indicates that innovative combinations can induce meaningful responses in *TP53*-mutated MCL. While these results are encouraging, many studies are still ongoing, and further data will be needed to determine their full impact in high-risk patients. Enrollment in clinical trials currently represents the most effective avenue to advance treatment for this population. Collectively, these findings confirm the central role of *TP53*, underscoring the importance of *TP53* testing with standardized, sensitive, and cost-effective tools to guide risk-adapted therapeutic strategies from the diagnosis.

**Table 2:** The table reports the trials cited in the main text and the technologies adopted to detect the *TP53* status. In all, *TP53* played a prominent role in the resistance to treatment or progression of MCL, regardless of the method applied for its identification.

Clinical Trials	*TP53* Mutations and\or Deletion Assessment	Treatment Resistance
European MCL Younger trial [30]	RQ-PCR	High-dose chemotherapy and autologous stem cell transplantation
Nordic MCL2 and MCL3 [10]	IHC, NGS	cytarabine, rituximab, and autologous stem-cell transplant (ASCT)
Korean, Multicenter, Retrospective Analysis [31]	IHC	Bendamustine and rituximab (BR)
TRIANGLE [32]	IHC	Ibrutinib in addition to chemoimmunotherapy
SHINE and ECHO [33,34]	IHC	Ibrutinib or acalabrutinib in addition to chemotherapy
BoVen [36]	IHC, NGS	Zanabrutinib, obinutuzumab, and venetoclax
SYMPATICO [37]	NGS	Ibrutinib combined with venetoclax
VR-BAC [38]	FISH, Sanger, NGS	Venetoclax in high-risk subgroups
ZUMA2 [39]	NGS, ddPCR	Brexucabtagene autoleucel
TARMAC [40]	FISH, NGS	CAR-T in combination with ibrutinib
NP30179 and NCT03075696 [42,43]	IHC	Glofitamab

## 3. Immunohistochemistry and Fluorescence In Situ Hybridization to Detect TP53 Point Mutation and Aberrations: Strengths and Limitations

Immunohistochemistry (IHC) is a technique commonly used to localize the presence of specific antigens in a tissue. In MCL, the lymph nodes or extra-nodal sites are removed and embedded in the formalin-fixed paraffin (FFPE) blocks for diagnostic purposes. The expression of p53 can be inserted in the diagnostic panel routinely applied for MCL diagnosis, which includes at least Ki-67, SOX11, CD20, and cyclin D1. Indeed, the European MCL Network (EMCL) recommends applying IHC to identify *TP53* expression due to the easy execution of the technique [11,44], although this approach has several pitfalls. IHC has high interobserver variability, a risk of cross-reactions [45], low sensitivity in detecting small amounts of protein mutation, and the inability to identify nonsense mutations. Regarding missense mutations, they may accumulate in the tumoral cell, allowing for their detection through IHC [17]. This procedure is currently well accepted in other types of cancers [46,47] and could be considered an effective surrogate for the laboratories that cannot perform targeted sequencing. Finally, IHC may lose the signal of truncating mutations, which represent 10–25% of all mutations [48].

It is acknowledged that the reproducibility and the standardization of the quantification of the image analysis in IHC could be challenging. The reproducibility of Ki-67 and p53 assessments was investigated by involving different hematopathology laboratories. Although the results were promising, it was highlighted that the major limitation in interpreting the results was related to the analyses of the samples performed by highly specialized pathologists under controlled conditions, which does not reflect routine diagnostic practice [44]. Computer-based IHC analysis using specific software, such as QuPath [49] or HALO [50], is able to quantify the antibody signals using continuous measurements of positive cells, and dichotomizing at a previously defined threshold; such computer image analysis overestimates results [51], but remains more precise than classic IHC, and could promote greater standardization and reproducibility of results.

In specific cases, the investigation of *TP53* gene alterations with IHC could be supported by Fluorescence In Situ Hybridization (FISH) to identify p53 aberrations. FISH could detect cytogenetic abnormalities such as amplifications, deletions, translocations, or chromosomal abnormalities [52]. The employment of FISH on FFPE is challenging due to the possible cellular overlapping and signal attenuation caused by nuclear truncations and heterogeneity of the tumoral region [17]. Furthermore, FISH entails more expensive stains than IHC, requiring specialized personnel to analyze the stained-tumoral sections [17]. In clinical practice, FISH is applied to detect the deletion of the p arm of chromosome 17 (del17p), which contains the *TP53* gene. This aberration is commonly correlated with adverse behavior in MCL [53]. The prognostic predictivity of the 17p deletion seems lower than *TP53* mutations themselves in several studies [30,38], both in nodal and nnMCL [17].

IHC and FISH represent practical and expedient initial diagnostic approaches for stratifying MCL patients. However, the low sensitivity, lack of standardization, and tissue requirements limit their clinical appeal when compared with molecular assays. For this reason, the combined use of IHC and high-throughput analysis may provide a more informative and precise approach for identifying high-risk patients.

## 4. Comparison Between Sanger and Next Generation Sequencing: Strengths and Limitations

Sanger sequencing is the first sequencing technology developed by Friedrich Sanger in 1977 [49]. This method is based on the capillary electrophoresis of individual fluorescently labeled sequencing reaction products [50]. Otherwise, NGS is a high-throughput technology able to simultaneously sequence millions of DNA fragments, providing comprehensive insight into the genomic mutational landscape of cancers [51], which could use DNA derived from tumoral tissue biopsies, blood, or body secretions [52].

The advantage of using Sanger sequencing is the high accuracy in detecting chromosomal abnormalities such as rearrangements, aberrations, clones, and hotspot mutations of a specific gene of interest. However, it presented several limitations, such as low throughput (one fragment per reaction), the ability to detect variants present in >15–20% of DNA, and it is not suitable for comprehensive genomic analysis, such as genome or exome sequencing [54,55]. Nowadays, Sanger sequencing is preferentially adopted as a validation method of specific genomic results derived from massive sequencing. For instance, Eskelund et al. performed Sanger sequencing to confirm the presence of *TP53* mutation in a small number of previously sequenced samples [10]. Another recent application in MCL was performed by Khouja et al., which verified the translocations and clonal IGH-V-(D)-J rearrangements [56]. Overall, Sanger sequencing is preferentially used to validate and confirm the presence of TP53 mutations and deletions (or further gene mutations or translocations of interest) in samples with low NGS coverage outcomes.

NGS highlighted the strong impact of specific genes, including *TP53*, on prognosis and treatment resistance [17,57,58] by determining a comprehensive genomic landscape of MCL. The significant advantage of identifying clonal (VAF >10%) and subclonal (VAF 2–5%) mutations allows for integrating clinical data, such as B-symptoms, MIPI score, and line of treatments, with a multitude of mutational data, which may predict worse outcomes and potential mechanisms of resistance, like the co-existence of del *CDKN2A* together with *TP53* mutations [17,59].

The challenges related to NGS are the high cost, the need for highly trained professionals, and the lack of standardization of the platforms in use. In an effort to standardize the procedure, the EuroClonality-NGS working group created the EuroClonality (EC)-NDC assay, which is able to detect clonal immunoglobulin (IG) and T-cell receptor (TCR) rearrangements, chromosomal translocations, copy number alterations (CNAs), and somatic nucleotide variants (SNVs). It was validated in multiple European centers [60]. In particular, it was employed on cohorts from the European MCL network (NCT00209222 and NCT00209209) [56]. Through the EuroClonality (EC)-NDC assay, the accuracy and sensitivity in trial MCL0208 molecular targets (not detected by Sanger sequencing and PCR) were successfully identified [61]. Another platform frequently adopted by the centers is the Cancer Personalized Profiling by Deep Sequencing (CAPP-Seq). It is prevalently used for the detection of circulating tumor DNA (ct-DNA) in plasma for MRD in order to monitor the follow-up of the disease, and track clonal evolution [58,62,63].

However, these two extremely valuable assays require a multistep workflow, making their application difficult to use in a clinical practice routine. Indeed, the data analysis is complex, time-consuming, and requires specialized biologists and bioinformaticians to prepare the samples and analyze the genomic outcomes. To address this limitation, a targeted Next-Generation Sequencing (tNGS) approach could be employed. For instance, Lorraine M. de Haan et al. employed tNGS to evaluate *TP53* alterations, demonstrating that IHC is an unreliable surrogate for accurately assessing *TP53* mutational status when compared to molecular profiling through tNGS [64]. Notably, the targeted approach was associated with significantly lower costs and shorter turnaround times compared to comprehensive NGS panels, thereby enhancing its feasibility for routine clinical implementation. Indeed, this strategy may be valuable in the research of *TP53* single-nucleotide polymorphisms (SNPs), which could have a higher incidence in the prognostication, monitoring, and prediction of disease progression and treatment resistance. Sequencing technologies vary widely in their approaches and depth of analysis. The choice of the most suitable method should be guided by several factors: (I) the availability and type of starting material (tissue sections, peripheral blood, or bone marrow), (II) the purpose of the study (diagnostic versus research), (III) overall costs.

## 5. Could Droplet-Digital PCR Be a Valuable Tool in Detecting TP53 Hotspots in the Hematological Field?

Droplet-digital PCR (ddPCR) is a molecular technique not widely used in clinical practice; it involves partitioning the sample into sub-reactions that are counted as either negative or positive by applying an analysis method based on Poisson statistics [65]. In comparison to NGS, ddPCR is not able to sequence the whole gene, such as Sanger sequencing, but it is useful to identify hotspots, sub-clonal mutations, and CNVs of the gene of interest with high sensitivity (VAF 0.01–0.1), reproducibility, rapidity, and at lower costs [65,66]. The use of this tool has multiple evidences in solid cancers, while in hematological malignancies, its potential has not been fully understood, even though there is evidence of its application in leukemias, lymphomas, myeloma, and chronic myeloproliferative neoplasms, as well as in the transplant field [65].

Notably, the ddPCR could also support the monitoring of MRD [17]. For instance, Chen L. et al. detected 29 different mutations of *TP53* in a cohort of patients during CAR-T treatment. Interestingly, the patients with the *TP53* mutation presented shorter OS and PFS in comparison with those without mutations [67].

These results suggest the importance of following the disease over time and the great efficacy and sensitivity of ddPCR in identifying pathogenic targets, such as *TP53*, compared to other high-throughput technologies.

However, in MCL, its use remains largely confined to research settings, where ddPCR is mainly applied as a validation tool (similar to Sanger sequencing) or for the detection of sub-clonal mutations in longitudinal studies, which are relatively few and typically involve small patient cohorts.

## 6. Finding the Right Method: Key Considerations

The implications of *TP53* in the progression and treatment resistance of MCL are evident, regardless of the VAF or type of mutation (nonsense, frameshift, or missense).

The IHC is the technique most commonly performed. The detection of p53 is commonly referred to as an overexpression of the protein, while the absence may be related to the complete deletion of p53. The presence of p53 overexpression is commonly associated with high-risk patients. However, it is important to note that the lack of p53 overexpression on IHC does not necessarily exclude the presence of an underlying *TP53* mutation, as some mutations do not result in protein accumulation and may therefore remain undetected by IHC. Therefore, to identify such cases and accurately define the type of mutation, sequencing and ddPCR are essential tools, as they allow precise characterization of *TP53* alterations and assessment of the VAF.

To confirm this result, it is recommended to investigate at the molecular level the presence of *TP53*. Indeed, sequencing and ddPCR allow us to define the type of mutation and the VAF. Typically, clonal mutations (VAF > 15–20%) are considered those with a greater impact on the disease’s aggressiveness, while subclonal mutations (VAF < 10%) are crucial in longitudinal studies to determine if treatment pressure affects them [56,64,68]. The assessment of the VAF and the type of mutation (LOF or GOF) may also help clinicians in the treatment choice. Moreover, the application of highly sensitive techniques, such as ddPCR and tNGS, could facilitate the implementation of *TP53* genomic knowledge and its implications in a longitudinal landscape.

**Figure 2:**
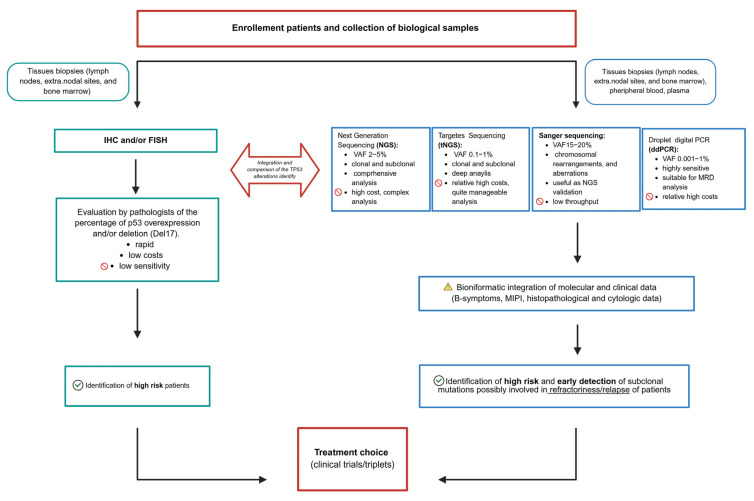
Flow chart summarizing key *TP53* detection strategies and their sensitivity, with an emphasis on their implications for clinical decision-making and guidance on method selection based on the available biological material.

Ultimately, a clear initial set-up and a well-defined objective—starting from patient enrollment—are essential, as the biological material available remains the main constraint in selecting the optimal technique for *TP53* detection. Choosing the right approach is therefore key not only to identifying high-risk patients but also to clarifying the biological and clinical significance of *TP53* mutations in MCL (Figure 2).

## 7. Conclusions and Future Perspectives

The involvement of *TP53* in the progression and treatment resistance of MCL patients is highlighted by multiple clinical studies, which report its importance in the prognostic evaluation and therapy management. Indeed, patients with *TP53* mutations generally have poor outcomes with conventional therapies. Nevertheless, durable responses remain challenging, making enrolment in dedicated clinical trials essential to provide access to innovative treatments and improve the management of these patients. Therefore, early *TP53* screening is becoming increasingly necessary to improve risk stratification and guide personalized treatment approaches, while promoting participation in clinical trials whenever possible. However, in clinical practice, the *TP53* screening is almost completely lacking due to the high cost and absence of specialized staff.

At the moment, IHC is the technique that is prevalently adopted to obtain results quickly and at low costs, although its accuracy could be poor. Moreover, the sequencing is predominantly performed in retrospective studies to characterize the genomic landscape of the cohort of interest, and only a few studies have performed molecular analysis routinely or in prospective studies due to the high costs. The future premises to overcome these issues could be to preferentially perform tNGS and ddPCR to obtain an accurate result about the *TP53* status in a short time and at a lower cost.

In conclusion, it is crucial to improve *TP53* detection in research and diagnostic laboratories and implement mutual protocols to improve standardization, accuracy, and reliability of the tests. Future integration of *TP53* testing into risk-adapted MCL management algorithms could optimize therapeutic outcomes and enable precision oncology. Indeed, *TP53* is no longer a marginal biomarker, but a true game changer in the management of MCL.

## Figures and Tables

**Table 1:** The table summarizes the main characteristics of the techniques commonly used to detect *TP53* mutations and aberrations. These methods differ not only in accuracy and sensitivity, but also in cost and in the type and depth of information they provide.

Techniques	Accuracy	Sensitivity (VAF)	Quantification	Costs	*TP53* Detection
Immunohistochemistry (IHC)	Low	-	Qualitative evaluation manual or automatic (QuPath/HALO)	Low	Protein localization (overexpression, missense variant, truncating variant, wild type)
Fluorescence In Situ Hybridization (FISH)	Intermediate	≥5–10%	Manual with fluorescence microscope or automated with imaging software	Intermediate High	Deletion 17p
Sanger sequencing	Intermediate/High	15–20%	Not quantitative	High	Mutation validation, and research of known targets
Next Generation Sequencing (NGS) and targeted NGS (tNGS)	High	2–5% and 0.1–1% (the sensitivity depends on the coverage adopted)	Quantitative	High Intermediate	Somatic nucleotide variations (SNVs), germline mutations, clonal evolution, and copy number variations (CNVs)
Droplet Digital PCR (ddPCR)	Very high	0.01–0.1%	Absolute quantification	Intermediate	Somatic nucleotide variations (SNVs), copy number variations (CNVs), and monitoring MRD

## Data Availability

No new data were created or analyzed in this study.
